# Using an Interaction Parameter in Model-Based Phase I Trials for Combination Treatments? A Simulation Study

**DOI:** 10.3390/ijerph18010345

**Published:** 2021-01-05

**Authors:** Pavel Mozgunov, Rochelle Knight, Helen Barnett, Thomas Jaki

**Affiliations:** 1 Department of Mathematics and Statistics, Lancaster University, Lancaster LA1 4YF, UK; rochelleknight96@gmail.com (R.K.); h.barnett@lancaster.ac.uk (H.B.); thomas.jaki@mrc-bsu.cam.ac.uk (T.J.); 2 MRC Biostatistics Unit, University of Cambridge, Cambridge CB2 0SR, UK

**Keywords:** dose-escalation, combination study, modelling assumption, interaction

## Abstract

There is growing interest in Phase I dose-finding studies studying several doses of more than one agent simultaneously. A number of combination dose-finding designs were recently proposed to guide escalation/de-escalation decisions during the trials. The majority of these proposals are model-based: a parametric combination-toxicity relationship is fitted as data accumulates. Various parameter shapes were considered but the unifying theme for many of these is that typically between 4 and 6 parameters are to be estimated. While more parameters allow for more flexible modelling of the combination-toxicity relationship, this is a challenging estimation problem given the typically small sample size in Phase I trials of between 20 and 60 patients. These concerns gave raise to an ongoing debate whether including more parameters into combination-toxicity model leads to more accurate combination selection. In this work, we extensively study two variants of a 4-parameter logistic model with reduced number of parameters to investigate the effect of modelling assumptions. A framework to calibrate the prior distributions for a given parametric model is proposed to allow for fair comparisons. Via a comprehensive simulation study, we have found that the inclusion of the interaction parameter between two compounds does not provide any benefit in terms of the accuracy of selection, on average, but is found to result in fewer patients allocated to the target combination during the trial.

## 1. Introduction

Traditionally, Phase I oncology trials predominantly investigated dose-finding for a single cytotoxic agent. In recent years, however, more complex dosing regimens are routinely considered. In particular combinations of drugs can have better therapeutic outcomes than a single anti-cancer drug treatment [1]. In a Phase I dual-agent combination trials, the aim is to determine the highest acceptable combination of doses known as the maximum tolerated combination (MTC) which is defined as the combination with probability of a dose-limiting toxicity (DLT) closest to the pre-specified target θ.

Single agent trials typically assume that toxicity increases monotonically with dose (also known as monotonicity assumption [2]) and hence there is only one target dose, the maximum tolerated dose (MTD). In combination trials, however, the monotonicity assumption typically does not hold for all combinations under study. This can be examplified where for the one level we have two drugs at two different doses, but the next level corresponds to an increase in one of the drugs but a decrease in the other. Here, the ordering of the toxicity probabilities is typically not known. The situation where the orderings between some dose combinations are known where others are not, is known as partial ordering [2]. A number of novel dose-finding design for combination trials were proposed [3,4]. The majority of novel designs are model-based and rely on a parametric model to fit the combination-toxicity relationship given the accumulating data. Typically, these models have between 4 and 6 parameteres [5,6,7] to be estimated given a small number of observations in the study. There is, however, an ongoing discussion as to whether a model with more parameters can be adequently fit in the setting of small sample sizes (usually between 20 and 60 in total and as few as two when the model is fit for the first time).

Specifically, Cunanan et al. [8] found that early phase dose finding trials studying toxicity and efficacy together often relied on copula models to specify the joint distribution of toxicity and efficacy, which include an additional correlation parameter that can be difficult to estimate. This is because of the small sample size in early phase trials. It was also found that a simple model that assumes independence between toxicity and efficacy performs just as well due to difficulty in estimating the copula model correlation parameters from binary data.

Similar reasoning to that of modelling the interaction between toxicity and efficacy as independent is thought to be relevant for models used in combination trials. Specifically, the question translated into whether an interaction term between the two agents could be estimated on small samples. Mozgunov et al. [9] have recently proposed a model-free dose-finding design for combination studies that assumed no-interaction and was found to perform more accurately in the considered setting than a model-based partial ordering continual reassessment method (POCRM) [10] and an alternative model-free design, PIPE [11]. However, one potential drawback of not including an interaction term in the combination-toxicity model is that less flexible models can collapse in some situations. While it was not found to be the case for the model-free design [9] due its flexibility, the question of the trade-off between not capturing unexpected combination-toxicity relationships and the challenging estimation of additional parameters still stands for the model-based combination designs.

In this work, we investigate for one particular model, namely the 4-parameter logistic model by Riviere et al. [6], the effect of removing specific parameters from the dose combination-toxicity model. We propose two model modifications reducing the number of parameters to three. For a fair comparison in the presence of no prior information on the compounds, we also propose an extensive calibration procedure to find so-called operational prior parameters, parameters that results in high accuracy in a range of different scenarios. Using the operational prior for each model considered, we explore the trade-off between estimation and flexibility via a compherensive simulation study in a number of clinical plausible scenarios with various interaction mechanisms of the compounds.

## 2. Model-Based Dose-Finding Design

### 2.1. Setting

Consider a dual-agent Phase I clinical trial. Assume that there are *J* dose levels of agent 1 (indexed by *j*) and *K* dose levels of agent 2 (indexed by *k*). A combination of the *j*th dose of agent 1 and the *k*th dose of agent 2 is denoted by (j,k). Let $Y_{ijk}$ be a Bernoulli random variable indicating whether a DLT occurs in patient *i*(i=1,…,N) given combination (*j*,*k*) where $Y_{ijk}=0$ if no DLT is observed in patient *i* and $Y_{ijk}=1$ if a DLT is observed. Let $\pi_{j,k}$ denote the toxicity probability given combination (j,k) and the logit function is defined as $logit\left(\pi_{j,k}\right)=\log\left\{\frac{\pi_{j,k}}{1-\pi_{j,k}}\right\}$. It is assumed that the dose-toxicity relationship increases monotonically with the dose-levels of each agent. The aim of the trial is to determine the MTC, the highest dose combination with a probability of toxicity closest to the corresponding target level of toxicity θ.

### 2.2. 4-Parameter Logistic Model

Riviere et al. [6] proposed the following logistic parametric form to model the combination-toxicity relationship:

$$Y_{ijk}∼\mathrm{Bernoulli}\left(\pi_{jk}\right)$$

(1) $$\mathrm{Model}\;M0:\;\;logit\left(\pi_{j,k}\right)=\beta_{0}+\beta_{1}u_{j}+\beta_{2}v_{k}+\beta_{3}u_{j}v_{k}$$

where $\beta_{0}$, $\beta_{1}$, $\beta_{2}$ and $\beta_{3}$ are unknown parameters that denote the intercept ($\beta_{0}$), the toxicity effect of agent 1 ($\beta_{1}$), agent 2 ($\beta_{2}$) and the interaction between the two agents ($\beta_{3}$), and $u_{j}=\log\left(\frac{p_{j}}{1-p_{j}}\right)$ and $v_{k}=\log\left(\frac{q_{k}}{1-q_{k}}\right)$ are the standardised doses of the two agents. The parameters $p_{1},\cdots ,p_{J}$, and $q_{1},\cdots ,q_{K}$, are the prior estimates of the toxicity probabilities for the dose levels of agent 1 and 2, respectively, when administered as monotherapy. The terms $u_{j}$ and $v_{k}$ are also known as the skeleton and are unchanged throughout the trial (with the estimation of the contribution of each agent to the combination toxicity being updated through the corresponding model parameters $\beta_{1},\beta_{2},\beta_{3}$).

Riviere et al. [6] defined the parameters $\beta_{0}$, $\beta_{1}$, $\beta_{2}$ and $\beta_{3}$ such that $\beta_{1}>0$ and $\beta_{2}>0$, ensuring that the toxicity probability is increasing with increasing dose levels of each agent alone; and $\forall \;k,\beta_{1}+\beta_{3}v_{k}>0$ and $\forall \;j,\beta_{2}+\beta_{3}u_{j}>0$, guaranteeing that the toxicity probability is increasing with increasing dose levels of both agents together; and the intercept $\beta_{0}\in R$. The model parameters are initialised by some prior distribution which describe beliefs about these parameters before any data are collected. We will refer to this original combination-toxicity model as “Model M0”.

Assume that $n_{j,k}$ patients are assigned to combination (j,k) and a total of $t_{j,k}=\sum_{i=1}^{n_{j,k}}Y_{ijk}$ toxic responses are observed for this combination. We define $N=(n_{1,1},n_{1,2},\ldots ,n_{j,k},\ldots ,n_{J,K})$ and $T=(t_{1,1},t_{1,2},\ldots ,t_{j,k},\ldots t_{J,K})$. Assuming some prior distribution of the model parameters $f(\beta_{0},\beta_{1},\beta_{2},\beta_{3})$ and applying Bayes Theorem, the posterior distribution of the model parameters is given by

$$f\left(\beta_{0},\beta_{1},\beta_{2},\beta_{3}|N,T\right)\propto \prod_{j=1}^{J}\prod_{k=1}^{K}\pi_{j,k}^{t_{j,k}}{\left(1-\pi_{j,k}\right)}^{n_{j,k}-t_{j,k}}f\left(\beta_{0},\beta_{1},\beta_{2},\beta_{3}\right)$$

where $f(\beta_{0},\beta_{1},\beta_{2},\beta_{3})$ is the joint probability density function of the prior distribution of the parameters. In the original proposal, independent prior distributions for each parameters were considered. We investigate the case of dependent prior distributions for the model parameters in Section 5.1.

For the parametric model and set of prior distributions of the model parameters, MCMC was used to obtain posterior samples from each distribution, that were then used to approximate the posterior distribution as they do not, generally, have a closed form. Specifically, Gibbs Sampling as implemented in the R-package rjags [12] was used. To satisfy the constraints of $\beta_{1}>0$ and $\beta_{2}>0$, the prior distributions defined on the positive real line were chosen (see Section 3 for further details). To ensure that $\forall \;k,\beta_{1}+\beta_{3}v_{k}>0$ and $\forall \;j,\beta_{2}+\beta_{3}u_{j}>0$, only those posterior samples of the model parameters that satisfy these constraints are taken forwards. Once the posterior samples for each parameter were obtained, they were used to get the posterior distribution of toxicity probability at each combination. These toxicity probabilities are subsequently used to govern escalation/de-escalation decisions (see Section 2.3).

### 2.3. Original Dose Finding Design

Riviere et al. [6] proposed to use model (1) in a sequential dose-finding trial with cohorts of patients sequentially assigned to different combinations based on accumulating evidence gained throughout the trial. Dose escalation and de-escalation was restricted to one level at a time (i.e., dose escalation and de-escalation along the diagonal is not allowed). The decision about which combination to assign to the next cohort was made based on the escalation/de-escalation constraints that were defined as follows.

Let $c_{e}$ be the probability threshold for dose escalation, $c_{d}$ the probability threshold for dose de-escalation and θ be the target toxicity probability. It is required that $c_{e}+c_{d}>1$. Let the current dose combination be (*j*,*k*) and {N,T} be the current data available on the number of patients thus far assigned to dose combinations and the number of DLTs observed, then

- If $P\left(\pi_{j,k}<\theta \left|N,T\right)\right)>c_{e}$, the combination dose level is escalated to an adjacent combination dose level {(j+1,k),(j,k+1),(j+1,k−1),(j−1,k+1)} and the next allocated dose combination (*j *${}^{\prime }$,*k* ${}^{\prime }$) is chosen such that it has a toxicity probability higher than the current value, ${\tilde{\pi }}_{j^{\prime },k^{\prime }}>{\tilde{\pi }}_{j,k}$, and a toxicity probability closest to θ
  $$\min_{j^{\prime },k^{\prime }}\{\left|{\tilde{\pi }}_{j^{\prime },k^{\prime }}-\theta \right|:\left(j^{\prime },k^{\prime }\right)\in \left(j+1,k\right),\left(j,k+1\right),\left(j+1,k-1\right),\left(j-1,k+1\right)\}$$
  If the current combination is the highest of the combination space, (*J*,*K*), the next cohort of patients will receive the same combination.
- If $P\left(\pi_{j,k}>\theta \left|N,T\right)\right)>c_{d}$, the combination dose level is de-escalated to an adjacent combination dose level {(j−1,k),(j,k−1),(j+1,k−1),(j−1,k+1)} and the next allocated dose combination (*j* ${}^{\prime }$,*k* ${}^{\prime }$) is chosen such that it that has a toxicity probability lower than the current value, ${\tilde{\pi }}_{j^{\prime },k^{\prime }}<{\tilde{\pi }}_{j,k}$, and a toxicity probability closest to θ. If the current combination is the lowest of the combination space, (1,1), the next cohort of patients will receive the same combination.
- If $P\left(\pi_{j,k}<\theta \left|N,T\right)\right)\leq c_{e}$ and $P\left(\pi_{j,k}>\theta \left|N,T\right)\right)\leq c_{d}$, the next cohort will be receive the current combination dose level.

Once the required sample size has been reached, the MTC is selected as the combination with the highest posterior probability of the risk of toxicity being within δ of the target probability, θ, $P(\pi_{j,k}\in \left[\theta -\delta ;\theta +\delta \right])$, and which has been used to treat at least one cohort of patients. Here, the parameter δ reflects how close the toxicity risk of a combination should be to the target in order for this combination to be considered promising.

For the purposes of this manuscript, we will focus on this specification of the design without stopping rules to ensure that the model specification is responsible for differences in the results. Each model can, however, also be used with stopping rules. For example, the most common stopping rule is to stop for safety—none of the studied combinations are deemed safe given the current data and the trial is terminated [13]. Furthermore, a design could stop once a given number of patients has been assigned to the same combination [14].

### 2.4. Alternative Models for the Combination-Toxicity Relationship

To ease the estimation problem of the combination-toxicity relationship with small samples, we explore a similar type of combination-toxicity relationship but with a reduced number of parameters. We consider modelling the drug combination-toxicity relationship based on the model with no interaction term, $\beta_{3}$,

(2) $$\mathrm{Model}\;M1:\;\;logit\left(\pi_{j,k}\right)=\beta_{0}+\beta_{1}u_{j}+\beta_{2}v_{k}$$

and the model with no intercept term, $\beta_{0}$

(3) $$\mathrm{Model}\;M2:\;\;logit\left(\pi_{j,k}\right)=\beta_{1}u_{j}+\beta_{2}v_{k}+\beta_{3}u_{j}v_{k}.$$

The model in Equation (2) will be referred to as “Model M1” and the model in Equation (3) will be referred to “Model M2”. Both models M1 and M2 use the same escalation and de-escalation criteria as outlined in Section 2.3 and all parameters within these models are as defined as before. Note that Model 2 resembles some of the features of the model by Thall et al. [5] but without the power parameters.

## 3. Choice of Prior Distribution

All combination-toxicity models described in the previous section employ a Bayesian paradigm for the sequential parameter estimation. Therefore, to initialise the model, prior distributions for each parameter should be defined before the trial commences. For each parameter, we use the same distributions as in the original work by [6]. In this section, using the example of the 4-parameter logistic design, we describe an algorithm for calibrating the hyper-parameters of the prior distributions of the model parameters $\beta_{0}$, $\beta_{1}$, $\beta_{2}$ and $\beta_{3}$. We will apply the same algorithm for all models considered in this manuscript.

The calibration of the hyper-parameters of the prior distributions for the model parameters will be performed through a grid search which consists of fitting a model for each of the possible combinations of hyper-parameters. The hyper-parameters of the models parameters—$\beta_{0}$, $\beta_{1}$, $\beta_{2}$ and $\beta_{3}$—will be calibrated using the following prior distributions:

(4) $$\beta_{0}∼N\left(0,a\right),\;\;\beta_{1}∼Gamma\left(b,b\right),\;\;\beta_{2}∼Gamma\left(c,c\right),\;\;\beta_{3}∼N\left(0,d\right)$$

for a∈A, b∈B, c∈C, and d∈D where A, B, C and D are the grids of values of hyper-parameters for each of the prior distributions. Following the original proposal, we use the normal prior for $\beta_{0}$ and $\beta_{3}$ centred at 0 to allow for the parameters to take both positive and negative values with neither being favoured. The Gamma prior is used for $\beta_{2}$ and $\beta_{3}$, to ensure that the parameters are positive, and are centred at 1 [6]. The latter implies that the toxicity effect of both agents is considered to be similar and that neither agent is favoured to increase the toxicity effect with increasing dose level more than the other.

While a model can be calibrated under all scenarios that are considered clinically plausible, such a procedure can become computationally infeasible if there are many scenarios to be considered. Therefore, we propose to perform a grid search using a subset of scenarios such that these would represent noticeably different (but still clinically plausible) combination-toxicity relationships. For example, one scenario with many doses having toxicity probabilities far below the MTC, and another where most doses have toxicity probabilities far above the MTC would reflect different extreme settings [14,15]. Additionally, some of the scenarios selected for the calibration should reflect various interaction mechanism between the two compounds. We provide examples of such scenarios in Section 4.

For the purposes of this manuscript, the models will be calibrated in terms of their accurate, namely, the proportion of correct selections (PCS). Let $\eta_{a,b,c,d}^{\left(h\right)}$ be the PCS under scenario *h* using prior parameters a,b,c,d. Then, the parameters $a^{⋆},b^{⋆},c^{⋆},d^{⋆}$ maximising the geometric mean of the PCS across *H* considered scenarios

(5) $$\max_{a\in A,b\in B,c\in C,d\in D}{\left(\prod_{h=1}^{H}\eta_{a,b,c,d}^{\left(h\right)}\right)}^{\frac{1}{H}}$$

are selected for the model. The use of the geometric mean over the arithmetic mean is proposed as a model performing uniformly across the considered scenarios is desirable. The geometric mean penalises poor results which is advantageous when looking at the ability of a model to consistently find the MTC across a variety of scenarios.

We would like to stress that the calibration procedure in this work is solely based on one measure (5) for the purposes of the comparing the models in terms of the MTC selections. At the same time, when considering calibration of the design hyper-parameters for a particular dose-finding clinical trial, some of the choices of hyper-parameters might not be considered as favourable even if they imply the highest accuracy. If there are some additional constraints to be imposed on the prior, this can be embedded within the procedure. For example, one can target the hyper-parameters such that the PCS is maximised but the prior toxicity probability at the lowest combination is not above 15%.

## 4. Numerical Evaluation

### 4.1. Setting

In this section, we study how the different parametric models for the combination toxicity relationship defined in Section 2 perform in a variety of clinically plausible settings. To investigate the design under each model, we simulate independent replications of Phase I trials that evaluate dual-agent drug combinations. We focus on the setting originally considered by Riviere et al. [6]. Specifically we use five dose levels for agent 1 and three dose levels for agent 2, resulting in 15 dose combinations to be investigated. The target toxicity is fixed at θ=0.3 and for each trial, an overall sample size of N=60 is used. A cohort size of 3 is used for all models and no stopping rules are used. For ethical reasons, each trial is started at the lowest dose combination (1,1). It was assumed in [6] that both agents were previously studied separately and intial guesses of toxicities for each dose of both agents administered as monotherapies can be provided by the clinical team. Specifically, for agent 1, the marginal initial guesses of toxicities, $p_{j}$, are specified as p=(0.12,0.2,0.3,0.4,0.5) and for agent 2, $q_{k}$ is specified as q=(0.2,0.3,0.4). These prior guesses are used to construct the skeletons $u_{j}$ and $v_{k}$ in Equation (1). The probability thresholds used to guide dose escalation and de-escalation follow [6] and are set at $c_{e}=0.85$ and $c_{d}=0.45$. The length around the target interval, δ is set at δ=0.1.

The objective is to evaluate the models in terms of their accuracy and the ethical counterparts: how many patients are assigned to overly toxic combinations and often is the target combination assigned during the trial. Specifically, we study the properties of the designs in terms of

- the percentages of correct selections (PCS) reflecting how accurately the design selects the target combination;
- percentage of patients allocated to a true MTC during the trial, reflecting the potential benefit the patients could get from efficient combination assignment;
- the percentage of DLTs observed throughout the trial reflecting how many patients suffer from the adverse events under each design.

### 4.2. Scenarios

Twenty scenarios are used to represent a variety of clinically feasible true underlying combination-toxicity relationships (Table 1). We focus on finding one MTC at the end of the trial, regardless of if there are multiple possible correct combinations within the scenario.

In Scenarios 1–7, the MTC is located along each diagonal of the combination space, moving from the lower (1,1) corner of the combination space in scenario 1 to the upper (5,3) corner in Scenario 7. As it is unknown at the planning stage what the true combination-toxicity relationship is, it is important that all these scenarios are used to ensure good operating characteristics across all these scenarios. Scenarios 2 and 6 each have two possible MTC dose combinations. Two more variants of each of these scenarios were added.

Scenarios 2 and 6 were altered by replacing, in turn, one of the two MTCs with a dose combination with a toxicity probability different to 0.3 whilst ensuring that the monotonicity assumption still holds to form the Scenarios 2.1, 2.2, 6.1 and 6.2. The Scenarios 1, 2.1, 2.2, 6.1, 6.2 and 7 represent extreme examples of the dose-toxicity relationship. For Scenarios 1, 2.1 and 2.2, they represent a steep combination-toxicity relationship with many of the doses higher in the combination space having toxicity probabilities far above the MTC. Comparatively, for Scenarios 6.1, 6.2 and 7, they show a flat combination-toxicity relationship where many of the doses lower in the combination have toxicity probabilities space are far below the MTC. Note that Scenarios 2.1, 2.2, 6.1, 6.2 also correspond to cases when one compound increases the toxicity of the combination more than the other—when increasing the dose of one compound leads just to the target toxicity of 30% but increase in another corresponds to an overly toxic (40%) dose combination.

Scenarios 8–12 were proposed by Riviere et al. [6]. In Scenarios 8, 10, 11 and 12 there are multiple MTCs which are not located along the same diagonal but throughout the combination space and in Scenario 9 there is one MTC located in the centre of the combination space. Furthermore, under Scenarios 8, 10, and 12, it is assumed that one of the compounds is more toxic than the other (i.e., the combination toxicity relationship is steeper in one compound). Scenarios 13 and 14 represent the scenarios with only one MTC located for high doses of one of the agents.

Under Scenario 15, all combinations are too toxic as the lowest combination already has the toxicity rate of 45%. In contrast, under scenario 16, all combinations are safe as the highest combination has a toxicity probability of 20%. Note that as the design does not include stopping rules, it is expected that a design with desirable properties would recommend the lowest and the highest dose in Scenarios 15 and 16, respectively.

Finally, Scenarios 11 and 14 were found to be well-approximated by the original 4-parameter logistic model but with the slope parameters for each agent being equal to $\beta_{1}=\beta_{2}=0$. This implies that the underlying combination-toxicity relationships are fully determined by the interaction parameter $\beta_{3}$ and these are the scenarios where one could expect to see the most gain in benefit in including the interaction term. Therefore, we will be using these 2 scenarios to assess further potential losses of not including the interaction parameter.

### 4.3. Calibration

The sets of values for the grid search for the hyper-parameters have been selected as

$$\begin{array}{cc} A= & \{0.1,1,10,100,200,400\},\\ B= & \{0.1,1,10\},\\ C= & \{0.1,1,10\},\\ D= & \left\{1,10,100,200,400\right\} \end{array}$$

for each of the considered models. Note that the values of a=10, b=1, c=1 and d=10 correspond to the prior distributions specified by Riviere et al. [6]. Therefore, these grids were chosen to explore lower and higher variance around the mean of the parameters compared to the originally considered prior distribution. Due to the computational costs, the hyper-parameters to be tried were chosen to be noticeably different from each other, e.g., at least increasing the variance of the parameter twofold. This aims at locating an approximately optimal (in terms of PCS) values. We will use these values to compare whether the proposed calibration procedure can provide benefits in terms of the operating characteristics. For each set of hyper-parameters combinations, we simulate 500 trials to evaluate dual-agent drug combinations.

As discussed above, the choice of scenarios for the calibration is crucial. As the calibration over all 20 scenarios would have been to computationally demanding, we specify a subset of four scenarios to reduce the compuational costs while still adequetly exploring the properties of the design specification under extreme scenarios. Specifically, Scenarios 2.1, 2.2, 6.1 and 6.2 from Table 1 are used for the calibration process. Scenarios 2.1 and 2.2 represent a steep combination-toxicity relationship with many of the doses higher in the combination space far above the MTC and Scenarios 6.1 and 6.2 show a flat combination-toxicity relationship where many of the doses lower in the combination space are far below the MTC. Importantly, we have selected scenarios with one MTC only as it was noted previously that model-based designs can strongly favour one of the MTC in scenarios with several of them. This undesirable favouring of particular combinations cannot be picked up via summary characteristics such as the PCS, and the inclusion of scenarios with one MTC only would mitigate this risk.

The results of the hyper-parameter calibration are given in Table 2.

For completeness, we also include the prior distribution originally proposed for the 4-parameter model (to which we refer as M0${}^{\left(0\right)}$) that will be further included in the simulation study. Note that the values of the hyper-parameters for M1 yielding the highest PCS were found to be on the bound of the selected grids of $\beta_{0}$ and $\beta_{2}$. We have further extended these grids to include a=400,500,600 and c=10,20,40 and it was found that indeed the hyper-parameters in Table 2 result in the highest PCS among the considered combinations of values (see Table 3).

Finally, the calibrated hyper-parameters choices imply various prior combination toxicity relationships, all of which could be plausible. For example, under Model M2, the prior point estimate is around 0.05% for the lowest combination (i.e., the starting combination is very safe) and the highest is nearly 40%. Such prior beliefs correspond to a sharp increase in toxicity on the 5th dose on one of the compounds. Then, for example, starting escalation at the lowest combination, if the earlier data would suggest that the highest dose is not as toxic as expected, the escalation to higher doses would be allowed.

### 4.4. Comparator: Optimal Benchmark for Combination Studies

While the primary goal of this work is to compare the performance of different models to each other, the similarity of the parameteric models defined (and the fact that all of the designs are model-based) could mean that all methods perform equally poorly on some scenarios. To provide a context for the comparison of operating characteristics, we include the performance of the non-parameteric benchmark for combination studies, a tool that provides an estimate for the upper bound on the PCS under the given combination-toxicity scenario [16]. The benchmark takes into account the “difficulty” of a scenario in terms of how close the toxicity risks for the combinations (under this scenario) to the target level of 30% are, and also accounts for the unknown monotonic ordering in the combination setting.

Specifically, at its first step, the benchmark utilises the concept of complete information [17], which assumes that the data for each patient given each dose is available (in contrast to the actual trials setting with this information for one dose only). Under complete information, the toxicity estimates at each combination are found. At the second step, the probabilities that these toxicity estimates come from various potential clinically feasible “orderings” of combination are found. These probabilities are assigned to the probability of each combination selection under the given ordering. We refer the reader to the recent work by Mozgunov et al. [16] for further technical details on the benchmark for combinations trials. We will refer to the benchmark design as “B”.

### 4.5. Results

A summary of operating characteristics using 4000 replications for all 4 models under Scenarios 1–14 with at least one MTC is given in Table 4. As some designs are expected to outperform others in some scenarios and perform worse in others, we also provide the geometric mean (GM) of the PCS and its variance (Var) across scenarios.

The calibrated model M2, the model with no intercept parameter, has a significantly lower average PCS of 37.0% compared to all other models. In 10 scenarios it has a PCS below 40% and has the lowest PCS amongst all models with a PCS of 11% in Scenario 13. The model M2 also has the lowest average percentage of patients allocated to a true MTC during the trial and the average percentage of DLTs throughout the trial is 32.8% which is the furthest from 30% compared to all other models. As this model is not performing comparably to all other models, it will not be considered further.

For model M0, which is the four-parameter model, two variations, with different set of prior parameters, are considered. The original model M0${}^{\left(0\right)}$ and the calibrated M0 have the mean PCS of 48.7% and 49.6%, respectively. Therefore, the use of a calibrated prior allowed to increase the average PCS by nearly 1% on average under all considered scenarios. Comparing the average performance across scenarios that were not included in the calibration (i.e., excluding 2.1, 2.2, 6.1, 6.2), the models perform comparably—within 0.4% for the average PCS. At the same time, the calibrated model results in a noticeably more consistent performance in terms of the PCS across the scenarios—the variance of the PCS is 232.6 for the original prior and 152.0 for the calibrated one. Furthermore, the calibrated model results in nearly the same proportion of patients allocated to the true MTC (difference of 0.3%) but with noticeably lower variance across scenarios—62.0 for the calibrated model against 105.6 for the original prior. The costs for a better and more consistent performance for the calibrated model M0 is having an average percentage of observed DLTs slightly above the target rate, 30.5%, but still close to the target toxicity. Therefore, the model with the calibrated prior results in a more consistent performance of the design, and therefore the model under this prior is taken for further evaluation with the competing models.

Model M1, the model with no interaction parameter, had the highest average PCS of 50.8% compared to the other models 1.2% higher that for the calibrated model M0. At the same time, M0 has slightly lower variance in the PCS across the scenarios of 152.0 compared to 165.2 for M1. In eleven scenarios the model M1 has either higher PCS than the model M0 or is within 3% of it (for 9 scenarios the same can be said for M0). Additionally, M1 allocated the highest average percentage of patients to a true MTC throughout the trial at 31.4% and has 1% lower average percentage of observed DLTs. We will now focus on comparing scenario-by-scenario performance as the two models M0 and M1 seem to perform somewhat comparably.

In scenarios with only one MTC, the two models show uneven performance depending on the location of the MTC. When the true MTC was located in either the lower (1,1) or higher (5,3) extremity or the centre (3,2) of the combination space, as in scenarios 1, 7 and 9 respectively, model M1 showed its best performances with PCS of 75%, 76% and 68% respectively. In all three of these scenarios the model M0 had a PCS at least 8% lower. Importantly, in scenario 1, the difference in PCS of 16% is observed between the two models in favour of the model M1. Scenario 1 shows a steep combination-toxicity relationship with all the doses higher in the combination space having a toxicity probability far above the MTC. Therefore, the significantly higher PCS for the model M1 is of particular preference in this scenario. The model M1 also allocates 56.1% of patients to the true MTC in scenario 1 which is the highest allocation across all scenarios compared to 41% for M0. The higher percentage of patients allocated to the true MTC for the model M1 suggests it is more conservative and less aggressive in its approach at allocating patients compared to the model M0. This is preferable, in particular in scenarios such as scenario 1 which shows such a steep combination-toxicity relationship. The most noticeable costs for this advantage of the model M1 is a loss of 18% PCS in scenario 12 with the target combination lying on various diagonals. The model M0 has a PCS of 58% compared to 40% for M1 that suggests that having a more flexible model (under the calibrated parameters) might be more beneficial under this scenario. Comparatively, in scenarios 2.1, 2.2, 6.1, 6.2, 13 and 14, both models show some of their poorest performances as these are the most challenging scenarios with a single MTC. It also confirmed by the benchmark that these scenarios are the most difficult—the benchmark results in its lowest PCS under these scenarios as well. In all these scenarios, both models have a PCS of 45% or lower.

Comparing the PCS in scenarios 11 and 14 which are approximately generated using the model with the intercept and interaction parameter model, $\beta_{1}=\beta_{2}=0$, one can find that the calibrated 4-parameter and 3-parameter with no interaction models perform within 3% of each other under scenario 11, and M1 outperforms M0 by 9% under scenario 14. Therefore, in the scenarios determined by the interaction only, the 4-parameter model does not provide any tangiable benefit and the 3-parameter model can approximate the combination-toxicity relationship well enough (or even better) to locate the true MTC.

Finally, comparing the performance of the models to the benchmark, as expected the benchmark results in the highest average PCS. Specifically, the ratio of the PCS compared to the benchmark, is 90% for M0${}^{\left(0\right)}$ and are 92 and 94% for M0 and M1, respectively. Furthermore, the benchmark results in the highest PCS under the majority of scenarios, in 11 out of 18 scenarious, the benchmark results in either higher PCS or within the simulation error. The lowest ratio of the PCS compared to the benchmark is nearly 44% for M0${}^{\left(0\right)}$ and around 71–72% for M0 and M1. In some of the scenarios, the models have shown to lead to super-efficiency [18], the phenomenon when the benchmark is outperformed. This can be explained by a design favouring particular combinations. The highest ratio of PCS compared to the benchmark is also achieved for M1 under Scenario 13–32% PCS for the benchmark versus 46% for M1 resulting in the ratio of 144%. This suggests that the design favours this combination under the calibrated hyper-parameters.

The model M1 assigned at least 30% of patients to a true MTC in more scenarios than M0. Of the two models, M0 was the only one in which for two scenarios—Scenario 9 and 13—the allocation was below 20%. For the model M0, the highest percentage of DLTs observed for all the scenarios was 38% in scenario 1 whereas for M1 this value is lower at 36%, also in scenario 1. This once again highlights that the model M0 is more aggressive in patients allocations. For the model M1, in six scenarios the percentage of observed DLTs lay in the interval [29%, 31%] compared to three scenarios for M0.

The results for scenarios 15 and 16 with no MTC are given in Table 5.

Under the overly toxic scenario 15, all of the models select the lowest combination with at least 92% with the minimum value of 92.7% for M2 and the highest of 98.9% for M1 (nearly 3% higher than for the calibrated model M0). Similarly, M1 allocated nearly 90% of patients to the lowest combination, which is the highest proportion among all models. Concerning, the safe scenario 16, M2 correspond to the poorest performance and selects the highest combination in nearly 80% compared to nearly 88% for both M0 models and nearly 94% for M1. The proportion of allocation is again the lowest for M2 and the highest for M1. Therefore, under both scenarios, Model 1 selects the closest to the target level combination with the highest probability and allocated more patients to the right dose.

As it was noted above, under the scenarios with several target combinations, model-based designs can favour particular combinations that will be reflected in uneven selection proportion of the target combinations. To explore this aspect of the considered models, we study the variance of each MTC selection within the scenario. Table 6 shows the variance between the percentage of selections of each possible correct MTC within the scenario for the models M$0^{\left(0\right)}$, M0, and M1.

All of the models show poor performance in evenly selecting between multiple MTC combinations across the scenarios. Comparing calibrated models, Model M1 has a lower average variance across these scenarios of 163.5 compared to 196.4 for M0. Despite having a lower average variance, the model M1 shows a greater range of values across the scenarios. In Scenario 8, M1 shows its highest variance of 1365.0. The model M0 has nearly the same range of variance across these scenarios, where its highest variance is 1336.4 in Scenario 11.

Overall, under the operational prior distributions calibrated to achieve the highest PCS under each parameter model, the model M1 without interaction parameter was found to have the best performance in the set of considered scenarios. The model M1 has the highest average PCS and has the greatest lowest ratio of the PCS compared to the benchmark across scenarios. The model M1 allocates the highest average percentage of patients to a true MTC throughout the trial. It has the closest average percentage of observed DLTs throughout the trial to the target value of 30%. The model M1 also has the highest proportion of MTC selections in the interval [0.2, 0.4] so is, on average, selecting combinations with toxicities around the target value of θ=0.3 more often. It has also demonstrated the most accurate performance in scenarios without the MTC. Furthermore, it containts one fewer parameter that can reduce the computations complexity of the proposed calibration procedure noticeably. One of the main drawbacks of model M1 is that it shows high variability when selecting the MTC when there are multiple possible MTCs in the combination space (e.g., Scenario 8).

## 5. Sensitivity Analysis: Joint Prior Distribution

### 5.1. Joint Prior Distribution

In the previous sections, following the approach of Riviere et al. [6], we have considered the parameters of the drug combination-toxicity relationship models to be independent. This assumption may not necessarily hold true and so we are interested in investigating whether considering the parameters to be dependent in a Phase I setting can improve the model’s performance. We therefore consider an alternative model that allows for dependence between the parameters in this section. Above, it was found that the calibrated three-parameter logistic model, M1, showed the best operating characteristics amongst the models considered and therefore we will use it her again but allow for a dependence structure between the three parameters $\beta_{0}$, $\beta_{1}$ and $\beta_{2}$ via a joint prior distribution with a given correlation structure.

We will model the joint distribution of the model parameters using a multivariate normal distribution. To ensure the conditions on parameters $\beta_{1}$ and $\beta_{2}$ to be positive, the following parameterisation will be used to

$${\left(\beta_{0},\;\beta_{1},\;\beta_{2}\right)}^{T}={\left(\tau_{1},\;\exp\left(\tau_{2}\right),\;\exp\left(\tau_{3}\right)\right)}^{T}$$

where the 3-dimensional random vector $\tau ={\left(\tau_{1},\tau_{2},\tau_{3}\right)}^{T}$ follows a multivariate normal distribution $\tau ∼N_{3}\left(\mu ,\Sigma \right)$ with mean vector μ and 3×3 covariance matrix Σ.

### 5.2. Parameters Calibration

To calibrate the joint prior, we use the same algorithm as before. The main difference being that now one needs to calibrate with respect to the correlation parameters. Specifically, using previously found calibrated values of the variance for the parameter $\beta_{0}$, we parametrise the covariance matrix of vector τ as

(6) $$\begin{array}{cc} \Sigma = & \left[\begin{array}{ccc} 400 & 20\rho_{0}\sqrt{M} & 20\rho_{0}\sqrt{n}\\ 20\rho_{0}\sqrt{m} & m & \rho_{1}\sqrt{mn}\\ 20\rho_{0}\sqrt{n} & \rho_{1}\sqrt{mn} & n \end{array}\right], \end{array}$$

(7) $$\begin{array}{cc} \mathrm{with}\;\mathrm{mean}\;\mu = & \left(0,-\frac{m}{2},-\frac{n}{2}\right) \end{array}$$

for $\rho_{0}\in P_{0}$, $\rho_{1}\in P_{1}$, m∈M and n∈N where $P_{0},\;P_{1}$ are sets of correlation values between the parameters and M,N are sets of hyper-parameters for the variance of the prior distributions of $\tau_{2}$ and $\tau_{3}$, respectively. To ease the computational burden, we have fixed the value of hyper-parameter corresponding to $\beta_{0}$ at the value found in Section 4.3, a=400 as the marginal distribution of $\beta_{0}$ is unchanged under this parametrisation. We have also assumed that $\mathrm{Corr}\left[\tau_{1},\tau_{2}\right]=\mathrm{Corr}\left[\tau_{1},\tau_{3}\right]=\rho_{0}$. This is based on the assumption that it is reasonable to assume that $\mathrm{Corr}\left[\beta_{0},\beta_{1}\right]=\mathrm{Corr}\left[\beta_{0},\beta_{2}\right]$ as the assumption that the correlation between the parameter of interaction, $\beta_{0}$, and the toxicity effects of agent 1, $\beta_{1}$; and the correlation between the parameter of interaction, $\beta_{0}$, and the toxicity effects of agent 2, $\beta_{2}$ is the same. The correlation between the two parameters $\tau_{2}$ and $\tau_{3}$ will be $\mathrm{Corr}\left[\tau_{2},\tau_{3}\right]=\rho_{1}$. As before, we require $\beta_{0}$ to be centred at zero.

As in the model M1${}^{\left(1\right)}$, we require the parameters $\beta_{1}$ and $\beta_{2}$ to be centred at 1 such that $E\left[\beta_{1}\right]=E\left[\beta_{2}\right]=1$. The parameters $\beta_{1}$ and $\beta_{2}$ both follow a log-normal distribution, hence, we take $\mu_{2}=E\left[\tau_{2}\right]=-\frac{m}{2}$ and $\mu_{3}=E\left[\tau_{3}\right]=-\frac{n}{2}$ to guarantee it.

To calibrate the covariance matrix, the following values of the hyperparameters were tried.

$$\begin{array}{cc} M= & \left\{0.5,1,1.3,1.4,1.5,1.6,2,2.5,5\right\}\\ N= & \left\{0.05,0.1,0.2,0.4,0.5,0.6,0.7,0.8,1\right\}\\ P_{0}= & \left\{-0.25,0,0.1,0.25,0.3,0.4,0.5,0.6,0.8\right\}\\ P_{1}= & \left\{-0.25,0,0.1,0.2,0.25,0.3,0.4\right\} \end{array}$$

Under the prior distributions in model M1, its hyper-parameter values correspond to m=1 and n=0.1 relating to the variance of $\beta_{1}$ and $\beta_{2}$; and $\rho_{0}=\rho_{1}=0$ as all the parameters in the model are independent. Higher values of *m* and *n* relate to higher variance which indicates a greater level of uncertainty for the parameters $\beta_{1}$ and $\beta_{2}$, respectively. Higher positive values of $\rho_{0}$ and $\rho_{1}$ reflect a stronger positive correlation between the two parameters in question, whilst lower negative values of $\rho_{0}$ and $\rho_{1}$ reflect a stronger negative correlation. The values of M and N on the grid were selected so that the variance of the model parameters could be both increased and decreased compared to the values in the model M1. The values of $\rho_{0}$ and $\rho_{1}$ were selected so that the parameters could be both positively and negatively correlated.

The final calibrated model M3 uses the hyper-parameter values m=1.6, n=0.5 and $\rho_{0}=\rho_{1}=0.3$ which corresponds to a weak positive prior correlation in the parameters. This combination of hyper-parameters provided the highest average PCS across the four calibration scenarios. This model will be referred to as M3.

### 5.3. Results

The model M3 was used in a large scale simulation study to assess its ability to determine the MTC under the scenarios in Table 1. The simulation study setting used was the same as described in Section 4.1. Again, we use 4000 replication to provide the results. We compare the model M3 to model M1. A summary of operating characteristics are given in Table 7.

The model M3 has the higher average PCS of the two models of 51.1% compared to 50.8% for model M1. Both models show a similar variance in PCS across the eighteen scenarios, however the variance is higher for the model M3 at 172.4 compared to 165.2 for M1. For 14 scenarios, the PCS for each model is within 3% of the other. In scenarios 2.1, 2.2, 6.1 and 12, the two models have PCS results that have a difference of greater than 3%. The model M1 has the higher PCS in scenarios 2.2 and 6.1. Both represent extreme examples of the dose-toxicity relationship where for scenario 2.2, many of the doses higher in the combination space are far above the MTC and for scenario 6.1, many of the doses lower in the combination space are far below the MTC. The largest difference in PCS between the two is in scenario 12 where it is possible to gain an additional 17% in PCS by selecting the model M3. At the same time, in scenario 6.1, model M3 has a PCS that is 10% lower than that of model M1. Comparing the performances to the benchmark, both model results in the ratio of average PCS of around 94%. Although, the lowest ratio of the PCS compared to the benchmark is nearly 71% for M1 under scenario 12 and 67% under scenario 6.1 for M3. Similarly to the findings for M1 above, super-efficiency can still be observed for M3, the ratio of the PCS is 138% under scenario 13 versus around 144% under the same scenario.

The model M1 on average allocates 31.4% of patients to a true MTC throughout the trial which is higher than for the model M3 which allocates 30.2%. In 14 scenarios the model M1 allocates a higher percentage of patients to a true MTC compared to only three scenarios for the model M3. In one scenario the allocation is the same. The average percentage of DLTs observed throughout the trial is highly similar for the two models at 29.6% for the model M3 compared to 29.4% for the model M1. In sixteen scenarios, the percentage of observed DLTs for each model was within 0.5% of the other and of these sixteen scenarios, the percentage of observed DLTs was the same for the two models in six scenarios.

Overall, for the operating characteristics we have studied, neither of the two models, M1 and M3, uniformly outperforms the other. The model M3 has a slightly higher average PCS so is able to locate a true MTC correctly more often under the scenarios investigated than the model M1. However, the model M1 allocates a higher average percentage of patients to a MTC throughout the trial, and allocates a higher percentage of patients to combinations with toxicity around the target value. The percentage of DLTs observed throughout the trial is similar for both models where the average values for each model is close to the targeted value of 30%. For both models, compromises in performance in one area needs to be made to achieve better performance in another.

## 6. Sensitivity Analysis: Different Sample Sizes

The results above concerned the setting with a sample size of up to N=60 patients enrolled in the trial. One can argue that the interaction parameter could be fitted more accurately with such a sample size but not with smaller sample sizes. Therefore, in this section, we study the behaviour of various models under different sample sizes under all considered scenarios.

Specifically, we will focus on the calibrated 4-parameter model M0, calibrated 3-parameter model M1, and calibrated 3-parameter model M3 with joint prior distribution on model parameters. These were found to result in similar operating characteristics and provide the most benefit in terms of the PCS while delivering safe designs. We consider 3 sample sizes, N=60 as before, and lower sample sizes N=48 and N=30 and fix the cohort size to be c=3 in each setting. Note that the values of prior parameters for each sample size will be the same for the given model, and these parameters were calibrated for N=60 and are given in Section 4 and Section 5.

**Figure 1:**
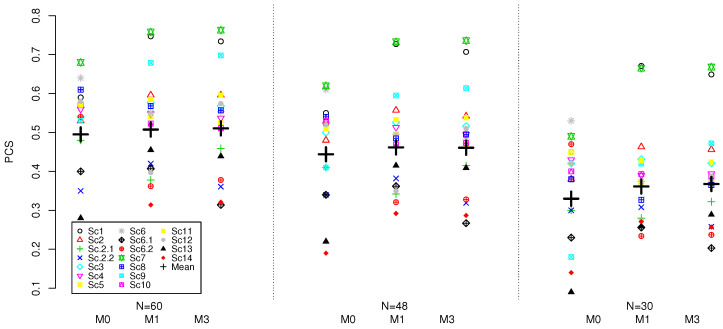
PCS for M0, M1 and M3 models under 18 scenarios and the sample sizes of N=60,48,30. Results are based on 4000 replications.

The results for the three model and various sample sizes based on 4000 replications are given in Figure 1.

Considering the PCS for each model individually, the lower sample size results in lower mean PCS for all models. The reduction in the sample size from N=60 to N=48 results in 5% mean PCS reduction for all three models. Further reduction from N=48 to N=30 results in additional nearly 9–11% for all models. Therefore, as expected, the sample size does have a major impact on accuracy of all model and larger sample sizes should be advocated for (regardless of the model used) in Phase I combination trials in order to achieve reliable recommendation of the MTC.

Comparing the models amongst themselves for a given sample size, a similar pattern as for N=60 discussed in previous section can be found. The 3-parameter models perform within 0.5%, on average, for all considered sample sizes and result in higher mean PCS compared to the M0 model: nearly 1% for N=60, nearly 2% for N=48, and approximately 3–4% for N=30. As a result, for lower sample sizes, the benefit provided by the models with fewer model parameters increases. Therefore, the interaction parameter does not seem to provide benefit in the mean PCS under the considered scenarios and, on the contrary, was found to result in lower average PCS as sample size decreases. The variance of PCS for all considered models under three considered sample sizes is similar across all three methods.

Considering other operating characteristics, the proportion of patients allocated to the MTC and the average proportion of the DLTs, all of the design performed similarly and the conclusion for N=60 stand. We refer the reader to the Supplementary Materials for the complete set of results for the lower sample sizes.

Overall, under the calibrated prior distribution targeting the highest PCS, the model with interaction resulted in 1–3% lower PCS across various sample sizes compared to the model with fewer parameters while resulting in nearly the same performance on other characteristics.

## 7. Discussion

In this work, we have conducted an extensive simulation study of various model-based dose-finding design for combination trials. Firstly, we have proposed a formal calibration procedure for the hyper-parameters under a given parametric curve to obtain an operation prior—the prior leads that leads to high PCS across many various scenarios. Applying the same procedure to each of the models allowed for a fairer comparison between models with various parameters. This is crucial as by definition, for the same hyper-parameters, a model with fewer model parameter would bare less uncertainty. We have shown that the proposed procedure allows for the improvement of the performance of the original 4-parameter model under the considered scenarios—it resulted in slightly higher average PCS yet noticeably more consistent performance across scenarios (reflected in the lower variance of the PCS).

Secondly, it was found that under the considered logistic-type models and the operational prior distributions calibrated to achieve the highest PCS for each model, the interaction parameters provided no benefit in terms of the PCS but resulted in lower ratio of the PCS compared to the benchmark than a model with the interaction term. The reduced number of parameters in the model has also had no negative effect on the performance under scenarios generated by the model with an interaction parameter only. Given a lower computations costs needed to calibrate fewer parameters, a logistic model with 3 parameters, the intercept, and a slope corresponding to each compound, is recommended for the consideration. We have also found that this recommendtion is consistent for various sample sizes, N=30,N=48,N=60. Importantly, this conclusion is obtained under the assumption when no reliable prior information on the interaction between the compounds (i.e., on $\beta_{3}$) is available. If, however, such information is available and is indeed correct, one can expect that the model with interaction parameter can have certain benefits.

Finally, comparing the 3-parameter models, specifying the joint prior distribution for the model parameters has not provided any tangiable benefit in terms of the PCS. However, it did result in lower computational costs and, therefore, could be considered in practice to speed up the calibration process proposed. Again, the conclusion is consistent across the various sample sizes.

Building on the computation aspect of the proposed calibration, it should be noted that the procedure is indeed quite computational costly given that it involves a grid search over each of the parameters. At the same time, the resulting hyper-parameters lead to good operating characteristics across many scenarios that enabled a more consistent aproach to the prior choice under the assumption of no prior knowledge about the compounds that is desired to be incorporated. If the calibration under various scenarios are parallised, it has found to result in feasible computation time. Moreover, the reader might use the parameters calibrated as the starting point in the the settings with similar numbers of doses of the compounds. At the same time, a less computationally demanding (yet reliable) procedure could be of interest and will be explored in the future.

In this work, we have focused on the comparison of designs that employ various parametric models for the combination-toxicity relationships but do not have any early stopping contraints. Constraints for stopping for safety (if all of the combinations are deemed unsafe) and stopping early for reaching a particular number of patients on one combination can easily be added.

Importantly, in this work, we have focused on a single form of the parametric curves for the combination-toxicity relationship—the logistic curve. While one could reasonably expect similar patterns under alternative parameter forms, this could be checked for a desirable model when taking a particular design forward in an actual trial. This could be done in a similar scheme to the simulation study as proposed in this manuscript. At the same time, the findings of this manuscript would warrant such an exploration.

Finally, the dual-agent setting is considered in this manuscript. However, combination studies looking at 3 and more agents could also be of interest. The extension of the proposed parametric model to more than 2 agents can be achieved via including the variables reflecting each compound. Then, the question will again be whether inclusion of the interaction terms can provide any benefits in terms of the operating characteristics and how these interaction should be included (e.g., only pairwise or should be interaction of 3 compounds be considered too). Given the findings of this work, the benefits of including interaction terms (that would complicate the estimation problem even further) should be scrutinised. It is also important to mention that the calibration procedure proposed for the setting more than 2 agents can be even more computational costly and might not be always feasible. For such cases, alternative (yet robust) approaches to the specification of the operational prior distribution should be studied.

## 8. Conclusions

Our study finds that carefully calibrated prior distributions can result in improved performance of the 4-parameter logistic model used for combination dose-finding. Moreover we show that only marginal benefits (if any) are seen when using an interaction term in the combination-toxicity model that are outweight by the additional complexity of the model.

## Figures and Tables

**Table 1:** Toxicity scenarios for the dual-agent combinations. The true MTC combinations are in bold.

	Agent 1									
Agent 2	1	2	3	4	5	1	2	3	4	5
	Scenario 1					Scenario 2				
1	**0.30**	0.40	0.50	0.60	0.70	0.20	**0.30**	0.40	0.50	0.60
2	0.40	0.50	0.60	0.70	0.80	**0.30**	0.40	0.50	0.60	0.70
3	0.50	0.60	0.70	0.80	0.90	0.40	0.50	0.60	0.70	0.80
	Scenario 2.1					Scenario 2.2				
1	0.20	**0.30**	0.40	0.50	0.60	0.20	0.40	0.50	0.60	0.70
2	0.40	0.50	0.60	0.70	0.80	**0.30**	0.50	0.60	0.70	0.80
3	0.50	0.60	0.70	0.80	0.85	0.40	0.60	0.70	0.80	0.90
	Scenario 3					Scenario 4				
1	0.15	0.20	**0.30**	0.40	0.50	0.10	0.15	0.20	**0.30**	0.40
2	0.20	**0.30**	0.40	0.50	0.60	0.15	0.20	**0.30**	0.40	0.50
3	**0.30**	0.40	0.50	0.60	0.70	0.20	**0.30**	0.40	0.50	0.60
	Scenario 5					Scenario 6				
1	0.05	0.10	0.15	0.20	**0.30**	0.03	0.05	0.10	0.15	0.20
2	0.10	0.15	0.20	**0.30**	0.40	0.05	0.10	0.15	0.20	**0.30**
3	0.15	0.20	**0.30**	0.40	0.50	0.10	0.15	0.20	**0.30**	0.40
	Scenario 6.1					Scenario 6.2				
1	0.01	0.03	0.05	0.10	0.20	0.03	0.05	0.10	0.15	0.20
2	0.03	0.05	0.10	0.15	**0.30**	0.05	0.10	0.15	0.20	0.40
3	0.05	0.10	0.15	0.20	0.40	0.10	0.15	0.20	**0.30**	0.50
	Scenario 7					Scenario 8				
1	0.01	0.03	0.05	0.10	0.15	0.05	0.08	0.10	0.13	0.15
2	0.03	0.05	0.10	0.15	0.20	0.09	0.12	0.15	**0.30**	0.45
3	0.05	0.10	0.15	0.20	**0.30**	0.15	**0.30**	0.45	0.50	0.60
	Scenario 9					Scenario 10				
1	0.02	0.10	0.15	0.50	0.60	0.05	0.12	0.20	**0.30**	0.40
2	0.05	0.12	**0.30**	0.55	0.70	0.10	0.20	**0.30**	0.40	0.50
3	0.08	0.15	0.45	0.60	0.80	**0.30**	0.42	0.52	0.62	0.70
	Scenario 11					Scenario 12				
1	0.12	0.20	**0.30**	0.40	0.60	0.04	0.06	0.08	0.20	**0.30**
2	0.20	**0.30**	0.40	0.50	0.67	0.10	0.20	**0.30**	0.50	0.67
3	0.42	0.52	0.62	0.70	0.80	**0.30**	0.42	0.52	0.70	0.80
	Scenario 13					Scenario 14				
1	0.05	0.08	0.10	0.15	0.20	0.01	0.03	0.06	0.10	0.20
2	0.10	0.15	0.20	**0.30**	0.40	0.04	0.07	0.12	0.20	**0.30**
3	0.20	0.40	0.50	0.55	0.60	0.08	0.10	0.20	0.40	0.50
	Scenario 15					Scenario 16				
1	0.45	0.50	0.55	0.60	0.65	0.01	0.02	0.05	0.10	0.15
2	0.50	0.55	0.60	0.65	0.70	0.02	0.05	0.10	0.15	0.17
3	0.55	0.60	0.65	0.70	0.75	0.05	0.10	0.15	0.17	0.20

**Table 2:** Calibrated prior parameters for each model and the corresponding PCS under 4 calibration scenarios. GM is the geometric mean of the PCS across the four scenarios. Results are based on 500 replications.

Model	$\beta_{0}$	$\beta_{1}$	$\beta_{2}$	$\beta_{3}$	Sc 2.1	Sc 2.2	Sc 6.1	Sc 6.2	GM
M$0^{\left(0\right)}$	N(0,10)	G(1,1)	G(1,1)	N(0,10)	-	-	-	-	-
M0	N(0,1)	G(1,1)	G(1,1)	N(0,100)	44.6	38.8	45.0	55.6	45.6
M1	N(0,400)	G(1,1)	G(10,10)	-	41.3	40.8	43.3	40.5	41.4
M2	-	G(1,1)	G(1,1)	N(0,100)	32.1	26.0	40.2	76.2	39.9

**Table 3:** Further calibration of M1: the geometric mean of the PCS across scenario 2.1, 2.2, 6.1, 6.2 for various values of hyper-parameters a=400,500,600, and c=10,20,40. The highest PCS is in bold. Results are based on 500 replications.

	c=10	c=20	c=40
a=400	**41.4**	38.2	39.2
a=500	40.7	38.9	37.1
a=600	37.9	39.3	37.6

**Table 4:** Percentage of correct selection in comparison of 5 dose-finding designs and the benchmark (B) under Scenarios 1–14. Percentage of patients allocated to a true MTC during the trial and percentage of observed DLTs throughout the trial. GM is the geometric mean and Var is the variance. In the top third of the table, the highest PCS and those within less than 3% across the dose-finding designs are shown in bold for each scenario. Results are based on 4000 simulations.

Sc	1	2	2.1	2.2	3	4	5	6	6.1	6.2	7	8	9	10	11	12	13	14	GM	Var
Percentage of correct selection (PCS)																				
M$0^{\left(0\right)}$	58	**59**	**56**	27	**58**	**56**	**56**	57	32	51	69	**64**	62	**62**	**61**	**61**	24	18	48.7	232.6
M0	59	53	48	35	54	**56**	**57**	64	**40**	54	68	61	53	57	57	58	28	22	49.6	152.0
M1	**75**	**60**	38	**42**	**58**	**55**	54	55	**41**	36	**76**	57	**68**	52	**59**	40	**46**	**31**	50.8	165.2
M2	47	37	30	26	35	41	47	**82**	**39**	**74**	57	28	33	39	43	36	11	23	37.0	296.6
B	82	61	49	46	57	54	57	61	46	42	83	65	58	53	50	56	32	41	53.8	166.1
Percentage of patients allocated to a true MTC during the trial																				
M$0^{\left(0\right)}$	45	45	38	17	42	36	34	36	23	24	38	36	24	44	38	40	14	16	30.9	105.6
M0	41	36	30	22	36	35	37	45	32	28	33	38	18	40	32	36	19	21	31.2	62.0
M1	56	40	21	32	38	34	31	36	25	21	48	35	37	33	34	26	19	22	31.4	91.8
M2	33	26	21	20	27	27	35	57	39	33	23	31	9	27	21	27	22	28	26.4	96.9
Percentage of DLTs throughout the trial																				
M$0^{\left(0\right)}$	37	33	35	36	30	28	26	24	23	25	20	28	33	30	32	30	28	26	28.6	21.1
M0	38	36	36	38	33	31	28	25	23	26	20	30	36	33	34	33	31	27	30.5	27.4
M1	36	33	34	34	31	29	28	25	25	27	21	29	31	31	32	32	30	27	29.4	13.2
M2	41	39	40	41	37	34	30	25	23	27	19	33	41	37	38	38	34	28	32.8	47.1

**Table 5:** Operating characteristics of the dose-finding designs under Scenarios 15 and 16. Results are based on 4000 simulations.

Sc	15	16
Selection Percentage of The Combination Closest to 30%		
M$0^{\left(0\right)}$	95.8	88.9
M0	96.1	88.3
M1	98.9	93.9
M2	92.7	79.8
Allocation Percentage To The Combination Closest to 30%		
M$0^{\left(0\right)}$	83.1	49.9
M0	78.1	45.4
M1	90.1	58.9
M2	61.7	31.3

**Table 6:** Variance between correct selections of MTCs in different locations throughout the combination space. GM is geometric mean. Results are based on 4000 simulations.

Scenario	2	3	4	5	6	8	10	11	12	GM
M$0^{\left(0\right)}$	18	250.7	157.9	233.8	269.1	5.1	245.9	1223.9	348.8	141.9
M0	33.4	244.5	140.1	234.0	257.6	35.7	294.3	1336.4	449.2	196.4
M1	22.4	234.5	200.5	417.0	8.9	1365.0	313.0	116.7	428.1	163.5

**Table 7:** Percentage of correct selection in comparison of 2 dose-finding designs and under the benchmark (B). Percentage of patients allocated to a true MTC during the trial and percentage of observed DLTs throughout the trial. GM is the geometric mean and Var is the variance. In the top third of the table, the highest PCS and those within less than 3% among the dose-finding designs are shown in bold for each scenario. Results are based on 4000 simulations.

Sc	1	2	2.1	2.2	3	4	5	6	6.1	6.2	7	8	9	10	11	12	13	14	GM	Var
Percentage of correct selection (PCS)																				
M1	**75**	**60**	38	**42**	**58**	**55**	**54**	**55**	**41**	**36**	**76**	**57**	**68**	**52**	**59**	40	**46**	**31**	50.8	165.2
M3	**73**	**60**	**46**	36	**57**	**54**	**52**	**55**	31	**38**	**76**	**56**	**70**	**51**	**60**	**57**	**44**	**32**	51.1	172.4
B	82	61	49	46	57	54	57	61	46	42	83	65	58	53	50	56	32	41	53.8	166.1
Percentage of patients allocated to a true MTC during the trial																				
M1	56	40	21	32	38	34	31	36	25	21	48	35	37	33	34	26	19	22	31.4	91.8
M3	56	38	24	26	36	32	30	35	20	20	48	28	36	31	33	31	21	21	30.2	90.9
Percentage of DLTs throughout the trial																				
M1	36	33	34	34	31	29	28	25	25	27	21	29	31	31	32	32	30	27	29.4	13.2
M3	36	33	35	34	31	30	28	25	25	27	21	29	31	31	32	32	30	27	29.6	14.2

## Data Availability

R codes implementing the considered designs are available on request from the corresponding author.

## Supplementary Materials

The following are available online at https://www.mdpi.com/1660-4601/18/1/345/s1. Complete set of results for the lower sample sizes.

## Abbreviations

The following abbreviations are used in this manuscript:

MDPI	Multidisciplinary Digital Publishing Institute
DOAJ	Directory of open access journals
TLA	Three letter acronym
LD	Linear dichroism
