# Supplementary material for: Using an Interaction Parameter in Model-Based Phase I Trials for Combination Treatments? A Simulation Study
Source: Int J Environ Res Public Health. 2021 Jan 5;18(1):345. doi: 10.3390/ijerph18010345 (PMC7796482; doi:10.3390/ijerph18010345)
Supplement: Supplementary file 1 [file ijerph-18-00345-s001.pdf]

Scenario	Sc 1	Sc 2	Sc 2.1	Sc 2.2	Sc 3	Sc 4	Sc 5
**M0**	44.9	52.5	43.8	43.7	52.5	54.3	56.5
**M1**	74.7	59.6	37.8	42.0	57.7	54.8	54.2
**M3**	73.4	59.6	45.9	36.1	56.8	53.7	52.4
							**Percenta**
**M0**	29.7	32.8	24.2	23.0	30.4	31.9	34.4
**M1**	56.1	39.8	21.1	31.7	37.6	34.1	31.0
**M3**	56.1	37.7	24.4	26.1	36.2	32.2	30.2
**M0**	24.4	21.9	22.8	23	20.3	18.8	17.3
**M1**	21.4	19.7	20.6	20.1	18.4	17.5	16.6
**M3**	21.5	19.9	20.7	20.4	18.7	17.8	16.8
**M0**	49.5	54.7	40.3	38.3	50.7	53.2	57.3
**M1**	93.5	66.3	35.2	52.8	62.7	56.8	51.7
**M3**	93.5	62.8	40.7	43.5	60.3	53.7	50.3

Scenario	Sc 1	Sc 2	Sc 2.1	Sc 2.2	Sc 3	Sc 4	Sc 5
**M0**	75.1	49.1	37.1	27.6	48.8	50.5	53.4
**M1**	72.7	55.7	34.2	38.2	52.6	51.3	49.4
**M3**	70.7	54.2	41.5	31.9	51.6	49.5	47.0
							**Percenta**
**M0**	48.3	33.0	21.4	22.5	30.6	31.2	30.6
**M1**	56.1	37.8	19.5	29.1	34.7	30.3	27.8
**M3**	56.4	36.2	22.8	24.0	33.3	29.6	27.1
**M0**	18.1	16.8	17.1	17.4	15.8	14.7	13.4
**M1**	17.2	15.6	16.4	16	14.5	13.6	12.7
**M3**	17.2	15.8	16.4	16.2	14.7	13.7	12.8
**M0**	37.7	35.0	35.6	36.3	32.9	30.6	27.9
**M1**	35.8	32.5	34.2	33.3	30.2	28.3	26.5
**M3**	35.8	32.9	34.2	33.8	30.6	28.5	26.7

Scenario	Sc 1	Sc 2	Sc 2.1	Sc 2.2	Sc 3	Sc 4	Sc 5
**M0**	22.5	40.7	30.6	32.7	39.6	40.3	41.9
**M1**	67.1	46.3	28.0	30.8	43.1	39.0	37.1

**M3**	64.9	45.6	32.2	25.8	42.1	39.4	37.9
							**Percenta**
**M0**	28.8	27.7	16.8	18.5	24.2	24.5	26.4
**M1**	57.6	33.0	17.0	22.7	29.4	23.7	20.2
**M3**	57.7	32.5	19.6	20.1	28.7	23.4	20.0
**M0**	12.5	10.8	11.8	11.7	9.6	8.4	7.2
**M1**	10.7	9.5	10.2	9.9	8.6	7.8	6.9
**M3**	10.7	9.5	10.1	10	8.6	7.8	7
**M0**	41.7	36.0	39.3	39.0	32.0	28.0	24.0
**M1**	35.7	31.7	34.0	33.0	28.7	26.0	23.0
**M3**	35.7	31.7	33.7	33.3	28.7	26.0	23.3

N=60								
**Sc 6**	**Sc 6.1**	**Sc 6.2**	**Sc 7**	**Sc 8**	**Sc 9**	**Sc 10**	**Sc 11**	**Sc 12**
**Percentage of correct selection (PCS)**								
61.7	43.8	44.5	69.0	55.5	64.4	59.0	55.6	53.1
55.1	40.7	36.2	75.8	56.8	67.9	52.3	58.5	39.7
55.4	31.4	37.8	76.3	55.7	69.8	51.1	59.6	57.4
**age of patients allocated to a true MTD during the trial**								
44.3	33.5	24.8	37.0	29.6	25.5	35.5	28.5	28.2
35.9	24.8	21.4	47.7	34.7	37.4	32.7	34.4	26.3
34.7	20.4	20.4	48.1	28.1	35.6	31.4	32.5	31.1
**Mean number of DLTs throughout the trial**								
15	14.3	16.2	12.1	18.4	20.6	19.8	20.7	20.5
15.2	14.9	16.1	12.7	17.5	18.4	18.3	19	18.9
15.2	14.8	16.2	12.7	17.5	18.8	18.5	19.4	18.9
**Percentage of DLTs throughout the trial**								
73.8	55.8	41.3	61.7	49.3	42.5	59.2	47.5	47.0
59.8	41.3	35.7	79.5	57.8	62.3	54.5	57.3	43.8
57.8	34.0	34.0	80.2	46.8	59.3	52.3	54.2	51.8

N=48								
**Sc 6**	**Sc 6.1**	**Sc 6.2**	**Sc 7**	**Sc 8**	**Sc 9**	**Sc 10**	**Sc 11**	**Sc 12**
**Percentage of correct selection (PCS)**								
54.5	19.6	51.5	70.9	45.4	67.0	45.7	54.2	54.8
49.5	36.2	32.1	73.3	48.5	59.5	46.9	53.3	35.0
48.3	26.7	32.8	73.6	49.5	61.4	47.3	53.8	51.0
**age of patients allocated to a true MTD during the trial**								
36.2	18.6	23.0	38.5	28.8	24.0	30.7	25.9	31.6
31.1	21.6	17.3	41.9	21.3	31.5	29.9	31.7	23.7
29.8	17.4	16.9	42.3	23.4	29.8	28.9	30.3	27.7
**Mean number of DLTs throughout the trial**								
11.6	10.9	12.4	9.4	14	15.6	15.3	16.3	15.3
11.5	11.2	12.3	9.5	13.5	14.5	14.2	15	14.8
11.5	11	12.3	9.4	13.5	14.7	14.4	15.2	14.7
**Percentage of DLTs throughout the trial**								
24.2	22.7	25.8	19.6	29.2	32.5	31.9	34.0	31.9
24.0	23.3	25.6	19.8	28.1	30.2	29.6	31.3	30.8
24.0	22.9	25.6	19.6	28.1	30.6	30.0	31.7	30.6

N=30								
**Sc 6**	**Sc 6.1**	**Sc 6.2**	**Sc 7**	**Sc 8**	**Sc 9**	**Sc 10**	**Sc 11**	**Sc 12**
**Percentage of correct selection (PCS)**								
49.7	28.3	38.4	53.1	32.9	35.3	43.7	40.3	36.9
37.4	25.6	23.4	66.5	32.7	42.0	39.3	42.8	27.3

37.2	20.3	23.7	66.8	36.5	47.2	38.7	42.4	38.8
**age of patients allocated to a true MTD during the trial**								
27.0	18.2	14.7	16.8	20.5	11.7	26.8	20.9	20.7
20.5	14.7	9.9	26.8	13.1	19.7	23.7	26.9	18.7
19.3	12.0	10.0	26.7	15.3	20.6	23.5	25.8	21.0
**Mean number of DLTs throughout the trial**								
5.8	5.2	6.3	4.4	7.7	9.4	9.1	10	9.3
5.9	5.6	6.4	4.7	7.3	8.5	8.2	8.9	8.5
5.9	5.5	6.4	4.7	7.3	8.5	8.3	9	8.4
**Percentage of DLTs throughout the trial**								
19.3	17.3	21.0	14.7	25.7	31.3	30.3	33.3	31.0
19.7	18.7	21.3	15.7	24.3	28.3	27.3	29.7	28.3
19.7	18.3	21.3	15.7	24.3	28.3	27.7	30.0	28.0

Sc 13	Sc 14	Geometric mean	Variance
36.9	33.1	50.5	89.8
45.5	31.4	50.8	165.2
43.9	32.1	51.1	172.4
19.5	26.0	29.4	34.6
18.8	22.2	31.4	91.8
20.6	20.7	30.2	90.9
18.6	16.2	18.7	10.8
17.7	16.4	17.6	4.8
17.7	16.4	17.7	5.1
32.5	43.3	**49.0**	**96.2**
31.3	37.0	**52.3**	**255.1**
34.3	34.5	**50.4**	**252.4**

Sc 13	Sc 14	Geometric mean	Variance
34.7	10.1	43.3	279.5
41.5	29.2	46.2	161.7
40.9	28.7	46.1	168.2
18.8	17.0	27.4	62.9
15.4	19.9	27.5	98.2
17.5	18.1	27.0	95.7
14.3	12.5	14.3	5.8
13.7	12.5	13.7	3.9
13.7	12.4	13.7	4.2
29.8	26.0	**29.8**	**25.1**
28.5	26.0	**28.5**	**16.8**
28.5	25.8	**28.6**	**18.0**

Sc 13	Sc 14	Geometric mean	Variance
19.9	23.2	35.0	80.4
25.9	27.1	36.1	160.2

28.9	25.7	36.8	156.8
12.1	15.2	19.9	30.2
9.6	14.8	20.4	119.2
10.6	12.7	20.3	117.6
8	6.2	8.2	5.6
7.5	6.4	7.7	2.8
7.5	6.4	7.7	2.8
26.7	20.7	**27.3**	**62.3**
25.0	21.3	**25.6**	**31.1**
25.0	21.3	**25.6**	**31.3**
